# Long-term forest soil warming alters microbial communities in temperate forest soils

**DOI:** 10.3389/fmicb.2015.00104

**Published:** 2015-02-13

**Authors:** Kristen M. DeAngelis, Grace Pold, Begüm D. Topçuoğlu, Linda T. A. van Diepen, Rebecca M. Varney, Jeffrey L. Blanchard, Jerry Melillo, Serita D. Frey

**Affiliations:** ^1^Department of Microbiology, University of MassachusettsAmherst, MA, USA; ^2^Department of Natural Resources and the Environment, University of New HampshireDurham, NH, USA; ^3^Department of Biology, University of MassachusettsAmherst, MA, USA; ^4^Marine Biological LabsWoods Hole, MA, USA

**Keywords:** climate change, microbial ecology, ribosomal RNA, rrn operon copy number, trophic strategy

## Abstract

Soil microbes are major drivers of soil carbon cycling, yet we lack an understanding of how climate warming will affect microbial communities. Three ongoing field studies at the Harvard Forest Long-term Ecological Research (LTER) site (Petersham, MA) have warmed soils 5°C above ambient temperatures for 5, 8, and 20 years. We used this chronosequence to test the hypothesis that soil microbial communities have changed in response to chronic warming. Bacterial community composition was studied using Illumina sequencing of the 16S ribosomal RNA gene, and bacterial and fungal abundance were assessed using quantitative PCR. Only the 20-year warmed site exhibited significant change in bacterial community structure in the organic soil horizon, with no significant changes in the mineral soil. The dominant taxa, abundant at 0.1% or greater, represented 0.3% of the richness but nearly 50% of the observations (sequences). Individual members of the Actinobacteria, Alphaproteobacteria and Acidobacteria showed strong warming responses, with one Actinomycete decreasing from 4.5 to 1% relative abundance with warming. Ribosomal RNA copy number can obfuscate community profiles, but is also correlated with maximum growth rate or trophic strategy among bacteria. Ribosomal RNA copy number correction did not affect community profiles, but rRNA copy number was significantly decreased in warming plots compared to controls. Increased bacterial evenness, shifting beta diversity, decreased fungal abundance and increased abundance of bacteria with low rRNA operon copy number, including Alphaproteobacteria and Acidobacteria, together suggest that more or alternative niche space is being created over the course of long-term warming.

## Introduction

Earth's climate is warming, and this is exacerbated by both biophysical (e.g., albedo) and biogeochemical [e.g., carbon (C) cycle] feedbacks (IPCC, 2013). Microbes are key players in every biogeochemical cycle, regulating greenhouse gas fluxes between soils and the atmosphere (Falkowski et al., 2008). Despite their pivotal role, we know little about how microbes respond to environmental change, and microbial dynamics are only beginning to be represented in ecosystem models (Reid, 2011; Treseder et al., 2012). Based on the improved predictive capacity of soil carbon cycling models when microbial physiology is considered, it is clear that microbes are important for understanding and predicting ecosystem processes (Allison et al., 2010; Li et al., 2014). New genomic approaches hint at what the most abundant organisms are, though their ecological roles are still unclear. A better understanding of microbial dynamics is critical for projecting the rate and magnitude of climate change (Melillo et al., 1990, 2002; Heimann and Reichstein, 2008).

At the Harvard Forest Long-Term Ecological Research (LTER) site, warming-induced disruptions to ecosystem C cycles have been observed as part of three ongoing warming studies. In the longest-running site (20 years, Table 1), soil respiration rates in the warming treatment were initially higher than controls, then slowed to become equal to controls after 12 years (Melillo et al., 2002). Most warming studies, including our own, have observed a short-term increase in CO_2_ emissions with warming (Rustad et al., 2001; Contosta et al., 2011; Wu et al., 2011; Davidson et al., 2012) as well as slowed CO_2_ emissions following this initial increase. Slowed CO_2_-C loss is concomitant with a depletion of labile C (Bradford et al., 2008; Frey et al., 2008; Melillo et al., 2011), as well as a decline in microbial biomass (Frey et al., 2008), thermal adaptation of soil microbes (Bradford et al., 2008) and a shift in microbial carbon use efficiency (Frey et al., 2013). More recently in our longest running (20-year) warming experiment, soil respiration has begun to increase again in warmed soils compared to controls. Partitioning the two components of respiration—microbial and root respiration—showed that on an annual basis, the majority of CO_2_ (70–80%) from forest soil plots was microbial regardless of heat treatment (Melillo et al., 2002, 2011). Climate warming and other cascading effects (such as drying) have been suggested to destabilize stored soil C in a manner that is dependent on organic matter age, protection, and other conditions that may alter microbial access and decay (Fierer et al., 2009; Schimel and Schaeffer, 2012). The non-linear response of soil respiration to warming, combined with the prior observations of microbial adaptation and that most soil respiration is microbial has lead us to the hypothesis that long-term warming has caused changes in the soil microbial community, which may be exacerbating C cycle feedbacks to the climate system.

**Table 1:** **Experimental sites that make up the warming chronosequence at Harvard Forest Long-Term Ecological Research (LTER) site**.

	**Soil Warming x N addition study (SWaN)**	**Barre Woods**	**Prospect Hill**
Start year	2006	2003	1991
Duration^a^ (years)	5	8	20
Number of replicate plots	6	1 megaplot with 25 subplots	6
Size of plots	3 × 3 m	30 × 30 m	6 × 6 m
Soil pH, O-horizon^b^	3.72	4.29	3.82
Soil pH, 0–10 cm mineral^b^	4.38	4.42	4.41
Total C (g C m^−2^), O-horizon^b^	3314 (404)	1772 (621)	2565 (247)
Total C (g C m^−2^), 0–10 cm mineral^b^	3478 (121)	1810 (92)	2859 (444)

a *Duration of the warming study is listed for the time of sample collection, in fall of 2011*.

b *Values listed are for control plot soils only*.

Studies that have examined both microbial community characteristics and activity as affected by warming, whether as lab experiments (Zogg et al., 1997; Frey et al., 2008) or field studies (Bardgett et al., 1999; Belay-Tedla et al., 2009; Wu et al., 2011; Zhou et al., 2012; Pold and DeAngelis, 2013) reveal that although the physiological response (e.g., CO_2_ flux) across experiments and biomes tends to be consistent in that warming increases C mineralization, the microbial community response is not, with microbial communities in different ecosystems responding differently to warming. Over the short term the net effect of warming on soil microbes tends to be increased microbial activity, including increased soil respiration (Rustad et al., 2001; Melillo et al., 2011; Wu et al., 2011). This response to warming is likely partly attributable to changes in the active fraction of the biomass, rather than changing community's constituents (Zogg et al., 1997; Andrews et al., 2000; Pietikåinen et al., 2005). A metaanalysis of 75 manipulative climate change experiments showed that not all soil microbial communities respond similarly to warming (Blankinship et al., 2011). Long-term experiments at the Kessler Farm Field Laboratory (KFFL) in the plains of central Oklahoma found increased diversity under warming and drought, suggesting that warming may have somehow “primed” the community to be more resilient and resistant to further disturbance (Zhang et al., 2005). Furthermore, Zhang and colleagues found that warming was associated with decreased net N mineralization and a significant shift in the substrate utilization profiles, indicating a change in the substrate availability for the community. Metagenomic sequencing on grassland soils from experimental plots that had been warmed continuously (+2°C) for 8 years showed that gene signatures for degradation of more labile C compounds—starch, chitin, hemicellulose, and cellulose—were activated, whereas genes involved in recalcitrant C degradation, such as lignin, were not stimulated by warming (Zhou et al., 2012; Luo et al., 2014). They concluded that warming in grasslands could result in a weakened positive feedback between the terrestrial carbon cycle and climate. While results from the early years of the Harvard Forest warming experiment are consistent with the grasslands study, the long-term effects point to a very different conclusion about climate feedbacks.

The Harvard Forest soil warming experiments offer a unique opportunity to understand how climate change affects soil microbial community composition over the course of long-term warming. This is important because bacterial activities are possibly involved in a positive feedback to climate (Melillo et al., 2002; Frey et al., 2008; Brzostek et al., 2012). Three forest plots experienced 5°C above ambient soil temperatures for 5 (SWaN Plots), 8 (Barre Woods) and 20 (Prospect Hill) years at the time of sampling, forming an experimental warming “chronosequence” (Table 1). Fatty acid methyl ester (FAME) analysis of the Prospect Hill warming experiment after 12 years of heating 5°C above ambient showed a significant decrease in fungal abundance and an increase in Gram-positive bacteria and Actinobacteria in warmed soils compared to controls (Frey et al., 2008). Previous results indicate observed changes in C source utilization (Frey et al., 2008), plant-microbial interactions (Butler et al., 2012), mass-specific microbial respiration rates (Bradford et al., 2008), and soil chemistry (Melillo et al., 2011, 2002). Together, these results suggest a substantial change in microbial substrate availability which would be consistent with changing bacterial community profiles and potential altered feedbacks to climate.

In this study, we used high-throughput sequencing of the V4 variable region of the 16S ribosomal RNA gene to test the hypothesis that bacterial communities have changed over two decades of simulated climate change in organic and mineral soil horizons. However, utilization of the ribosomal RNA gene for phylogenetic analysis carries the caveat that the rRNA operon often exists at multiple copies per genome, which both confounds estimates of genomic abundance by over-estimating taxa with multiple rRNA operon copies (Crosby and Criddle, 2003; Větrovský and Baldrian, 2013) but also suggests life strategy, where taxa with multiple rRNA operon copies have faster growth rates, while taxa with single copies tend to be associated with oligotrophic environments (Klappenbach et al., 2000; Stevenson and Schmidt, 2004). We estimated copy number by phylogenetic inference using the available complete genome sequences in GenBank (Kembel et al., 2012), and used this to examine the effect of copy number correction on community profiles. We also used these estimates to test the additional hypothesis that average bacterial copy number was decreased by long-term warming, which would be consistent with observations of microbial adaptation (Bradford et al., 2008) and decreased soil carbon with long-term warming (Melillo et al., 2011). Finally, we performed quantitative PCR (Q-PCR) of bacterial and fungal communities, as well as the phyla Actinobacteria, Acidobacteria, and class Alphaproteobacteria, to measure changes in absolute abundance of these dominant and dynamic groups.

## Materials and methods

### Field experiment

All three experimental sites at the Harvard Forest Long-Term Ecological Research (LTER) site (Petersham, MA) were established in mixed hardwood forest stands with dominant tree species being paper and black birch (*Betula papyrifera* and *lenta*), red maple (*Acer rubrum*), black and red oak (*Quercus velutina* and *rubra*), and American beech (*Fagus grandifolia*). The soils are coarse-loamy inceptisols. The warmed plots at all three sites have been heated continuously by the use of resistance heating cables buried at 10 cm depth in the mineral horizon (Melillo et al., 2002). Controls in the longest running experiment are disturbance controls (which had cables buried but never activated); effects of disturbance on microbial community composition (Frey et al., 2008), soil inorganic nitrogen, and carbon dioxide flux (Peterjohn et al., 1994) have been minimal. Heating was to 5°C above ambient, a temperature increase which falls within the range of worst-case-scenario model projections for increased global air temperature by the year 2100 as projected by the Intergovernmental Panel on Climate Change (IPCC, 2013). Mean monthly temperatures at Harvard Forest range from 19°C in July to −5°C in January and average annual precipitation is 112 cm, distributed relatively evenly throughout the year.

### Sample collection

Soil samples were collected October 25–27, 2011 from each of the three sites in the chronosequence with four replicates from each treatment at Barre Woods, Prospect Hill and SWaN (Table 1). Soil was collected from the organic horizon (which varies in depth from 1–5 cm) and the upper 10 cm of mineral soil in the heated and control plots of all three studies, with four replicates totaling 48 samples (3 sites × 2 warming treatments × 2 soil horizons × 4 replicates). The organic horizon was collected as intact 20 × 20 cm blocks to the depth of the mineral soil, and the underlying mineral soil was sampled to 10 cm depth in the same locations using a custom-made stainless steel auger (9 cm diameter). Subsamples of both horizons were taken and immediately flash frozen in the field and stored at -80°C until extraction.

### Nucleic acid extraction

Soils were extracted twice based on previously published methods (DeAngelis et al., 2010) with a few modifications. Frozen soils were extracted for DNA and RNA simultaneously (Griffiths et al., 2000) using modified CTAB extraction buffer (0.25 M phosphate buffer (pH 8), 5% hexadecyltrimethylammonium bromide (CTAB) in 1M NaCl) and 50 μl of 0.1 M ammonium aluminum sulfate (Braid et al., 2003). Three replicate extractions were performed for each sample, then pooled and put through the Qiagen All DNA/ RNA Mini kit (Qiagen, Valencia, CA).

### Amplification and sequencing

Library generation and sequencing of the V4 region of the 16S ribosomal RNA gene were performed as per a recently published protocol (Caporaso et al., 2012). The V4 region of the 16S ribosomal RNA gene was amplified on an Eppendorf ProS thermal cycler using the primers 515F and 806R, where the forward primers contained a subset of 48 of the 8 bp barcoded primers. Reactions were performed in a final volume of 25 μl using Takara ExTaq with 200 pM of each primer, 25 μg of BSA and 2 units of DNA polymerase (Takara Mirus Bio, Madison, WI) with 10 ng template per reaction. PCR amplifications were performed at 50°C annealing temperatures (Tm), with an initial denaturation (5 min) followed by 30 cycles of 95°C (30 s), Tm (25 s) and 72°C (120 s), and a final extension of 72°C (10 min). Triplicate amplification reactions were verified by agarose gel electrophoresis, pooled, then cleaned using Qiagen MinElute kit (Qiagen Sciences, Valencia, CA). Cleaned amplicon pools were quantified using picogreen and quality assessed by nanodrop. Sequencing was performed by the Molecular Biology Core Facility at the Dana-Farber Cancer Institute using standard operating procedures. Due to the low diversity of the library 50% by DNA mass PhiX spike was added to the pooled, barcoded sample just before running. Samples were sequenced using the MiSeq platform to generate 2 × 150 bp paired-end reads.

### Sequencing data analysis

Paired-end sequences were assessed for quality using FastQC (Andrews, 2010), and initial quality filtering and assembly was performed using FLASH using default parameters (Magoč and Salzberg, 2011). Sequences were then binned into operational taxonomic units (OTUs) and taxonomies assigned using the subsampled open-referenced OTU picking method in QIIME (Caporaso et al., 2010) at 99% sequence identity based on the October 2012 greengenes taxonomy (DeSantis et al., 2006), where the fasta reference file was truncated to include just the V4 region (Werner et al., 2012). Chimeric sequences were detected using UCHIME (Edgar et al., 2011; Wright et al., 2012). We removed sequences observed only once or twice (singletons or doubletons), as well as erroneous sequences that were probable chimeras (DeSantis et al., 2006). A second community matrix of dominant taxa was generated with only taxa present in the data set at 0.1% relative abundance or greater. Community matrices were rarefied to the number of taxa in the sample with the lowest number of observations for most analyses excluding diversity analyses, since there tends to be a positive correlation between richness and depth of sequence sampling. Sequences are available in GenBank under accession numbers SRP040706, BioProject ID PRJNA242868.

### Quantitative PCR

Total bacterial and fungal abundances were measured for all 48 samples by quantitative PCR (Q-PCR) using 341F (5′-CCT ACG GGA GGC AGC AG-3′) and 534R (5′-ATT ACC GCG GCT GCT GGC-3′) (Muyzer et al., 1993) primers for total bacteria and ITS1f (5′-TCC GTA GGT GAA CCT GCG G-3′) and 5.8 s (5′-CGC TGC GTT CTT CAT CG-3′) (Fierer et al., 2005) for total fungi. Standard curves were based on linear PCR product from *Klebsiella* sp. str. BRL6-2 for bacteria and *Saccharomyces cerevisiea* for fungi, after purification, quantification and dilution. All samples were run as technical duplicates on an Eppendorf Realplex2, using the QuantiFast SYBR Green PCR Kit (Qiagen) with 10ng of DNA per 25ul reaction and primers at a final concentration of 1uM. PCR program was 5 min 95°C initially, followed by 40 × (10 s 95°C, 10 s 58°C, 20 s 60°C), and melt curve (60°C–95°C). Efficiencies were 101.6% for fungal, with an R^2^ of 0.9923, and 107.8% for bacteria, with an R^2^ of 0.9972. Total Acidobacteria, Actinobacteria and Alphaproteobacteria abundances were measured for all eight Prospect Hill organic horizon samples by quantitative PCR (Q-PCR). Primer sets for Acidobacteria were Acid31 (5′-GAT CCT GGC TCA GAA TC-3′) and Eub518 (5′-ATT ACC GCG GCT GCT GG-3′) (Fierer et al., 2005); for Actinobacteria were Act920F3 (5′-TAC GGC CGC AAG GCT A-3′) and Act1200R (5′-TCR TCC CCA CCT TCC TCC G-3′); and for Alphaproteobacteria were α682F (5′-CIA GTG TAG AGG TGA AAT T-3′) and 908αR (5′-CCC CGT CAA TTC CTT TGA GTT-3′) (Bacchetti De Gregoris et al., 2011). To generate standard curves, we used *Micrococcus luteus* (ATCC 381), *Caulobacter crescentus* str. CB15N (Peter Chien, pers. comm.), and *Acidobacterium capsulatum* (DSMZ 11244) for Actinobacteria, Alphaproteobacteria, and Acidobacteria, respectively. These standard curves were based on PCR product except for Acidobacteria, which was based on plasmid. The identity of all organisms and specificity of amplified products were confirmed by Sanger sequencing. Actinobacteria had a two-step amplification cycle with 10 s at 95°C followed by 30 s at 61.5°C. Acidobacteria and Alphaproteobacteria had a three-step cycle with 10 s at 95°C, 20 s at 55°C, and 10 s at 60°C. Soil dry weights were obtained by drying 10 g fresh soil in a 105°C oven for 3 days, and abundances are reported as counts per dry weight of soil. Counts represent genome equivalents not considering the confounding factor of multiple small subunit ribosomal RNA operon copies per genome.

### Statistical analysis

The experimental design was a fully replicated design with three warming experiments: Prospect Hill (20 years warming), Barre Woods (8 years warming), and SWaN (5 years warming); one treatment of warming or no-heated control; two soil horizons (organic and mineral); and four field replicates, resulting in 48 samples total. For the copy number correction, we used a recently published method (Kembel et al., 2012), where the copy number corrected OTU table was generated using copy numbers estimated in R based on ancestral state reconstruction (Matsen et al., 2010). Weighted average copy number is based on multiplying relative abundance of OTUs by their estimated copy number, then calculating average copy number per sample. The UniFrac distance matrices were generated using FastUnifrac (Hamady et al., 2009). Beta diversity was estimated based on weighted Unifrac distance matrices used to generate principal coordinates plots, and Procrustes analysis was performed to compare principal component plots of copy number corrected environmental file and uncorrected environmental file. Evenness was measured by Pielou's J, and richness measured as total number of taxa. We used seven methods to identify indicator OTUs of warming in the Prospect Hill organic horizons: (1) indicator value analysis (Dufrêne and Legendre, 1997); (2) volcano plots using two as the minimal fold change, 0.05 as the p value threshold in a *t*-test adjusted for multiple comparisons completed in METAGENassist after normalizing the data using Pareto Scaling (Arndt et al., 2012); (3) Nearest Shrunken Centroid classification (NSC) (Tibshirani et al., 2003); (4) partial least squares discriminatory analysis; (5) Bayesian groupings; (6) rank mobility based on mean abundance and reporting the top 5% of OTUs which showed the greatest change in rank; (7) and paired Student's *T*-test using the Benjamini-Hochberg correction (Benjamini and Hochberg, 1995). To compare whether Q-PCR copies differed between warmed and control plots, we used Bayesian inference (Kruschke, 2013), because this method yields richer inference considering the relatively small sample size. All statistical analyses were performed in R using the RStudio interface (RStudio, 2012; R Core Team, 2014), including packages reshape (Wickham, 2012), vegan (Oksanen et al., 2011), ggplot2 (Wickham, 2009), limma (Smyth, 2004), pplacer (Matsen et al., 2010), indicspecies (De Cáceres and Legendre, 2009), pamr (Tibshirani et al., 2003), caret (Kuhn, 2008), phyloseq (McMurdie and Holmes, 2013), pls (Mevik and Wehrens, 2007), and BEST (Kruschke, 2013).

## Results

Sequencing produced 3,487,689 high-quality sequenced observations of the V4 16S ribosomal RNA gene region (Table S1, Figure S1). Sequencing depths before rarefaction ranged from 33,545 to 112,502 sequences per sample, with a mean of 63,293 reads and median of 62,838 reads. For most analyses, communities were rarefied to 33,545 sequences. Sequence reads were binned into operational taxonomic units (OTUs) based on 99% sequence identity (Werner et al., 2012), both because bacteria with nearly-identical 16S rDNA sequences may represent variable genotypes and different species (Suau et al., 1999; Hold et al., 2002; Konstantinidis and Tiedje, 2005) and because many functional traits are phylogenetically conserved up to 0.01% ribosomal RNA gene sequence dissimilarity (Martiny et al., 2013). After removal of singletons, doubletons and chimeras 2,938,751 observations remained (Table S1) that were binned into 45,875 OTUs, a scale of diversity on par with current estimates of soil bacterial diversity (Torsvik et al., 2002; Gans et al., 2005; Roesch et al., 2007).

### Microbial community responds to soil warming after 20 years

**Figure 1:**
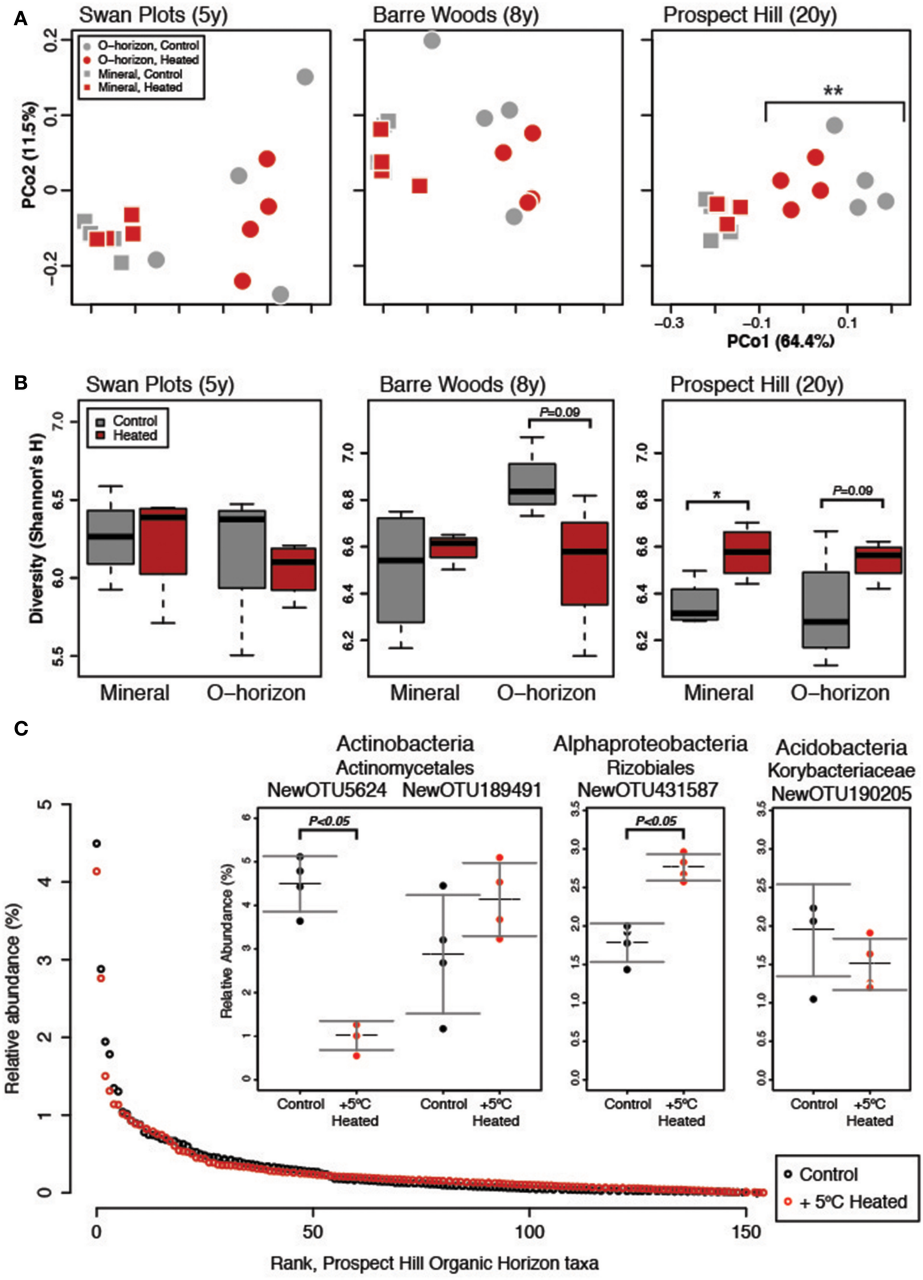
**Measures of diversity for the warming chronosequence, based on the dominant subset community (*N* = 155)**. **(A)** One PCoA ordination was performed on all sites, both treatments and both soil types, but the three sites are shown separately for clarity: SWaN Plots, Barre Woods, and Prospect Hill. **(B)** Diversity as measured by Shannon's H index is shown for the three sites as box (first and third quartiles) and whisker (95% CI) plots where the solid bar is median. **(C)** Rank abundance curve of the 155 dominant species in the community, with inset showing the three most abundant taxa and their relative abundance in heated versus control treatments averaged for the three sites. Statistical significance is indicated as ^*^P < 0.05, ^**^P < 0.01.

Soil warming had a statistically significant impact on bacterial community structure, but only after 20 years of warming. After 20 years, organic warmed soils were significantly different from those of control plots (Figure 1A). The sites warmed for 5 or 8 years showed no significant treatment effect on beta diversity, but bacterial communities in organic horizons warmed for 20 years began to resemble mineral soils. The strongest effect on bacterial community structure in our analysis was soil horizon (organic versus mineral), followed by site and then warming treatment (Table S2), with no significant interaction between soil horizon and treatment.

While soil warming affected community structure only in the organic horizon, diversity as measured by Shannon's H index was higher at the 20-year warmed Prospect Hill site (*P* < 0.01, Figure 1B, Table S3A), and was on average lower at the SWaN site compared to the other two sites (*P* < 0.001, Table S3B). In the Prospect Hill site, changes in diversity were driven more by increasing evenness (*T*-test *P* < 0.01; Bayesian effect size 1.60, 95% range −3.1 to −0.16) than changes in richness (*T*-test *P* = 0.06; Bayesian effect size −0.983, 95% range −2.05–0.20). The observed increase in bacterial diversity was driven strongly by decreased abundance of a few dominant taxa.

### Dominant subset reflects total community response to warming

The bacterial community was observed to be highly uneven: only 155 taxa (0.3% of total) present at 0.1% relative abundance or higher accounted for over half of the 3 million observations (Figure 1C). Comparison of the full community to the dominant subset community showed that the two were highly correlated (Procrustes *R* = 0.987, *P* < 0.001; Mantel test *R* = 0.980, *P* < 0.001). The same trends emerged for the dominant community as were observed for the full community, where soil horizon, then site, were major drivers of community structure, and that warming only affected the organic horizon of the 20-year long-term warmed soils (Table S2). This is perhaps not surprising considering that these dominant taxa, though comprising less than 1% of the richness, represented over half of all observations. The treatment effect on organic soil communities was stronger when looking at the dominant compared to the full community (Table S2), suggesting that dominant taxa were more strongly affected by warming.

The composition of the dominant subset community was similar to that of the total community, though the richness and phylogenetic range were much depleted. Compared to the 43,909 OTUs found in 33 phyla and 133 families in the total rarefied community, the dominant subset community was comprised of 155 OTUs found in 6 phyla and only 19 families. These included the phyla (and subphyla) Acidobacteria (57 OTUs), Alphaproteobacteria (40), Actinobacteria (25), Verrucomicrobia (11), Gammaproteobacteria (11), Planctomycetes (6), Firmicutes (2), Betaproteobacteria (2), and Deltaproteobacteria (1).

**Figure 2:**
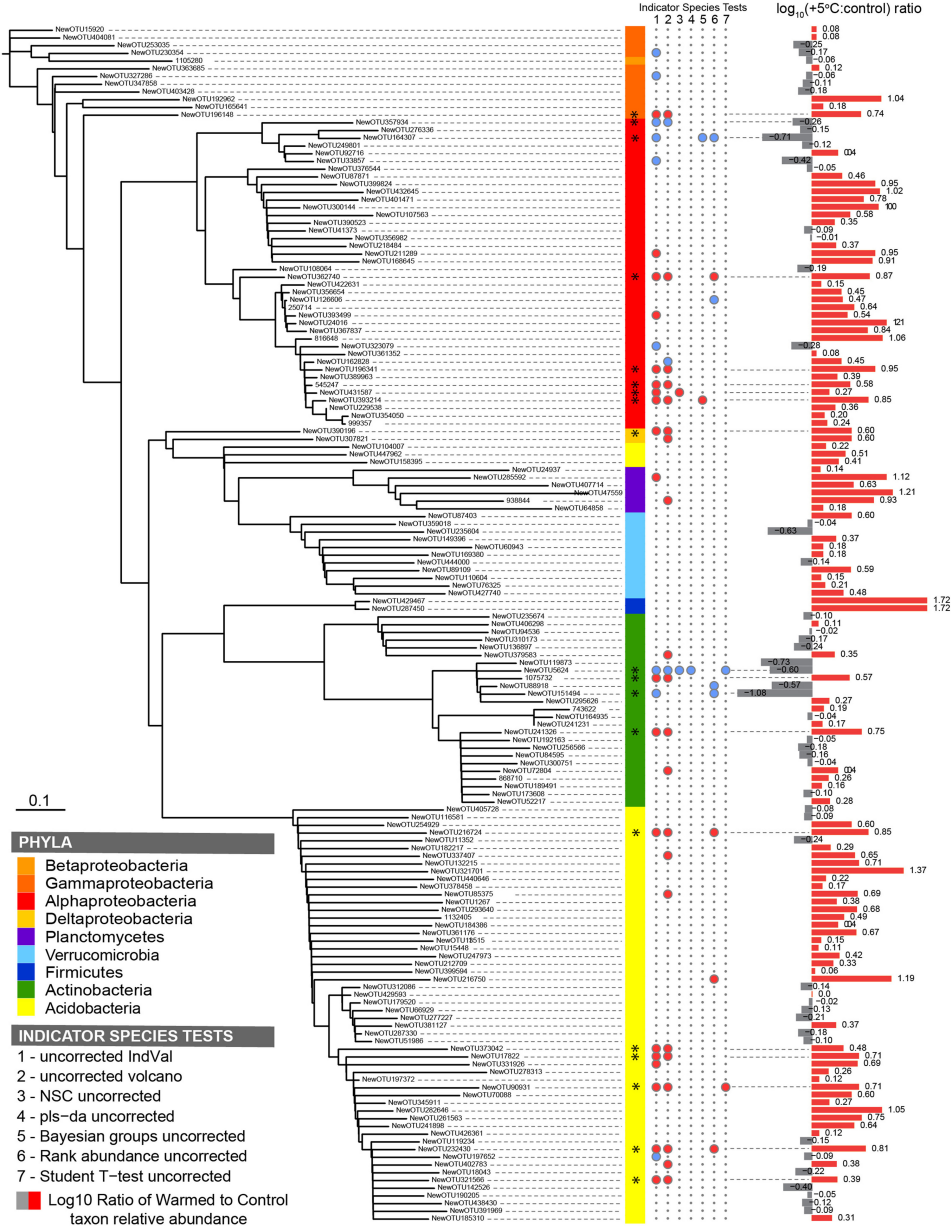
**Phylogenetic tree of the dominant subset community (*n* = 155) in the Prospect Hill 20-year warmed sites, where (from left to right) the color strips denote phylum-level classification; the circles denote a significant indicator species test (*P* < 0.05) with blue circles indicative of control treatments and red circles indicative of heated treatments; bars show fold change in OTU abundance with warming treatment**. The seven indicator species analyses are (1) Dufrêne and Legendre's IndVal, (2) fold change (volcano-plots), (3) nearest shrunken centroid, (4) PLS-DA loadings test, (5) Bayesian group comparison, (6) Rank abundance test, (7) Student's *t*-test. Taxa with two or more significant indicator species tests are marked (^*^).

**Figure 3:**
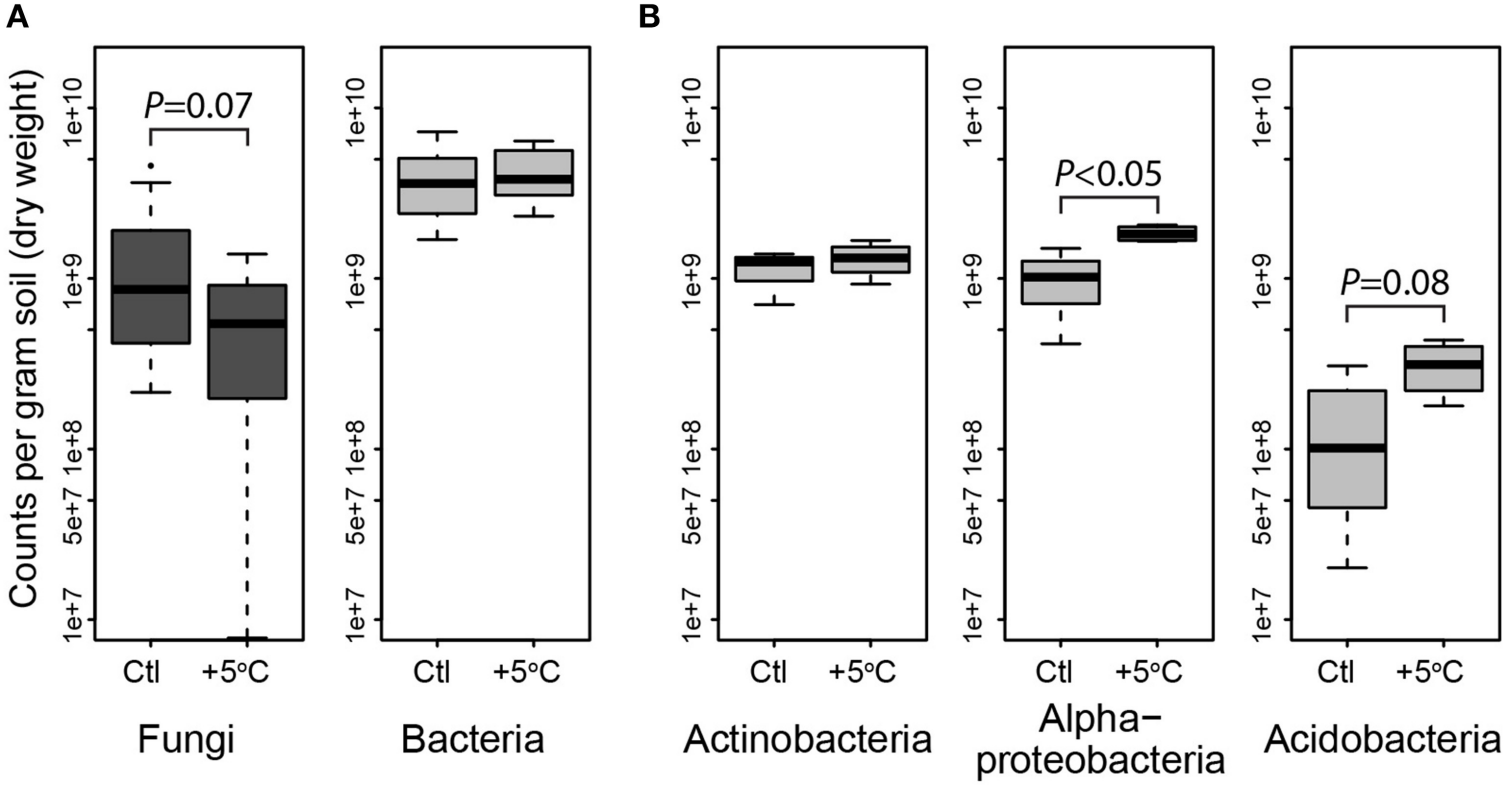
**Quantitative PCR of bacteria and fungi, and the phyla Actinobacteria, Acidobacteria and Alphaproteobacteria showing abundance of dominant microbial phylogenetic groups for both treatments in organic horizon (averaged by site, which was not a significant factor)**. Fungi were less abundant, and bacteria unchanged in heated (+5°C) compared to control (Ctl) organic horizon soils across the three sites **(A)**. In the organic horizons of the Prospect Hill site, Actinobacteria were unchanged, while Alpha-proteobacteria and Acidobacteria were enriched by long-term warming **(B)**. Means are shown as as box (first and third quartiles) and whisker (95% CI) plots where the solid bar is median.

Most bacterial taxa in the dominant subset responded positively to long-term warming (Figure 2), though we observed no change in absolute abundance of total bacteria with warming treatment by Q-PCR (Figure 3A, Table S4). In the organic soil horizon, bacterial abundance was unaffected by warming, while fungal abundance was marginally decreased with warming (Bayesian effect size 0.744; *t*-test *P* = 0.07). Power analysis showed that 6.5 biological (field) replicates per group would distinguish the treatment effect on fungi in the organic horizon (for power = 0.90, significance level 0.95). Fungal ITS Q-PCR counts in the mineral horizon were unaffected by warming. The Q-PCR data showed that there were more fungal rRNA operon copies in the organic horizon compared to the mineral soil (*P* < 0.05), with the same amount of bacterial rRNA operon copies in the two soils. Bacteria 16S ribosomal RNA gene counts outnumbered fungal ITS counts by an order of magnitude in the organic layer and mineral layers (*P* < 0.001 for both soils).

### Select taxa were especially sensitive to 20 years of warming

To identify the taxa responsible for the observed changes in the organic horizon bacterial community after 20 years of warming, indicator species analysis was performed using seven different methods (Table S5). Taxa that are more likely indicators of warmed or control conditions for the 20-year warmed site were defined as significant by multiple indicator species analyses (Figure 2). These analyses revealed that 19 of the 155 dominant taxa were differentially abundant in the organic soil horizon of the heated compared to control plots, with 15 increased in the warmed plots and four decreased. All six Acidobacteria detected as indicator species were indicators of heating treatment, with increased abundance in warmed compared to control soils. The four Actinobacteria were divided evenly between being indicators for heated or control treatments, and all belonged to the class *Actinobacteria* and order *Actinomycetales*. The last nine species were all Proteobacteria, and the two Proteobacteria that were indicators for control treatments were both Alphaproteobacteria in the order Rhodospirilales. The seven Proteobacteria that were indicators for warming treatments consisted of five Rhizobiales (Alphaproteobacteria), Syntrophobacterales (Deltaproteobacteria), and Xanthomonadales (Gammaproteobacteria). Measures of absolute abundance (based on Q-PCR) of Actinobacteria, Alphaproteobacteria and Acidobacteria were performed because taxa in these groups were dominant in our MiSeq observations: 15% Actinobacteria (25 of 155 OTUs), 26% Alphaproteobacteria (40 of 155), and 37% Acidobacteria (57 of 155). Absolute copies of Actinobacteria did not differ between warmed and control plots, though heated plots had more Alphaproteobacteria (*P* < 0.05) and trended toward having increased abundances of Acidobacteria (*P* = 0.08) compared to control plots in the Prospect Hill organic horizon (Figure 3B, Table S4).

### Mean ribosomal RNA copy number is significantly depleted by warming

**Figure 4:**
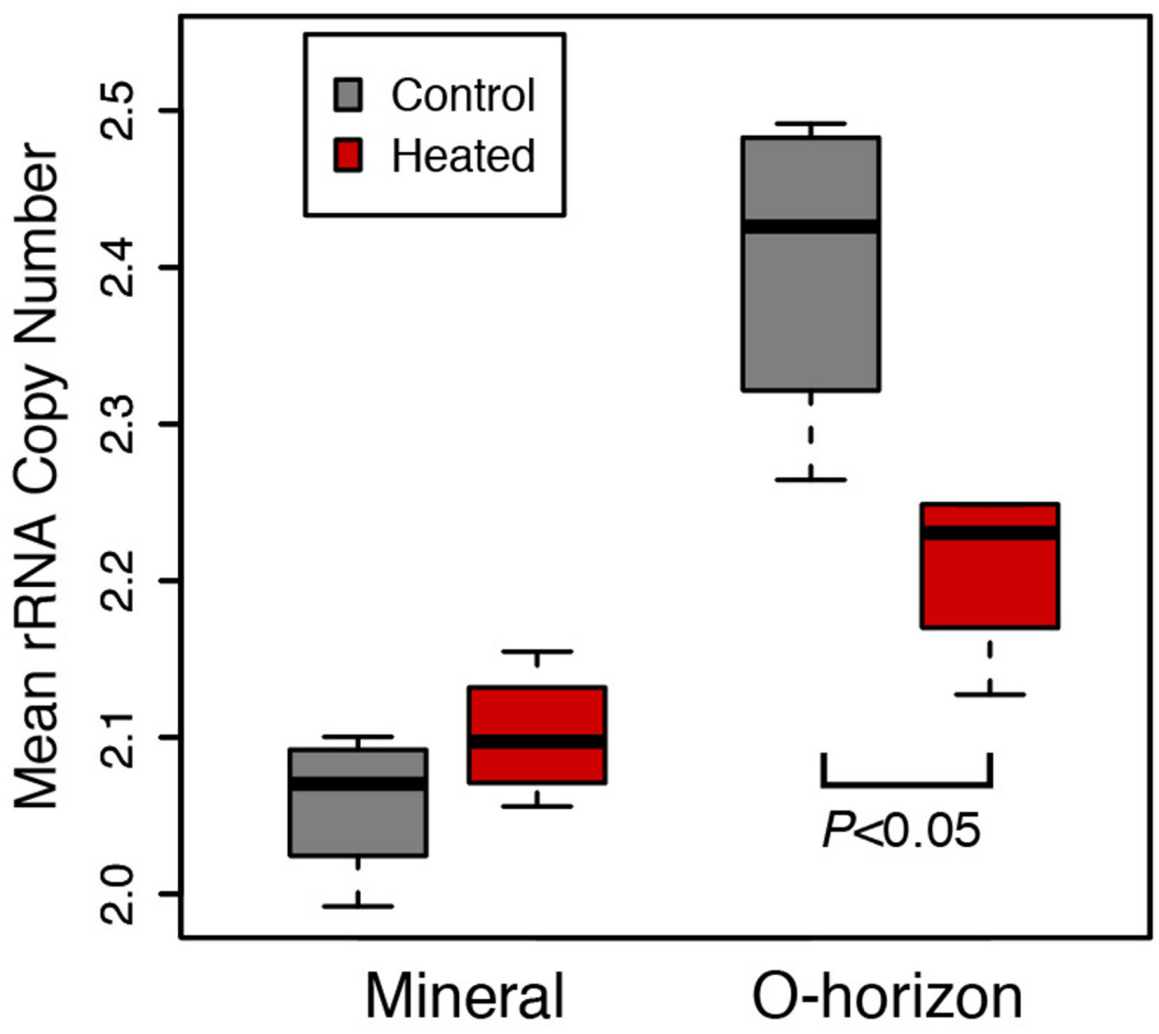
**Mean ribosomal RNA copy number was calculated for Prospect Hill (20 years warmed) soil communities, and these calculations were based on phylogenetic inference (see methods for details)**. Means are shown as as box (first and third quartiles) and whisker (95% CI) plots where the solid bar is median; statistical significance was determined based on ANOVA (*P* < 0.05).

The organic horizon in the 20-year warmed site (Prospect Hill) had lower average ribosomal RNA copy number of the bacteria present in heated compared to control soils (2.40 Control, 2.28 Heated, *P* < 0.05; Figure 4). Community structure did not change substantially when copy number was corrected based on Procrustes analysis of community profiles (Monte Carlo *P* < 0.05), with a high degree of similarity (*R* = 0.9511) between the copy number corrected and uncorrected PCoA community models (Figure S2). The most abundant taxa tended to have fewer than four copies of the 16S ribosomal RNA operon, while lower abundance taxa had as many as 14 estimated copies (Figure S3). Of the top 15 most dominant taxa, 13 had only one or two copies of the 16S ribosomal RNA operon. When copy number correction was applied, only 28 taxa changed rank such that they were no longer in the dominant subset community (comprised of 155 taxa).

## Discussion

Mounting evidence suggests that soil microbes play a role in elevated CO_2_ emissions and soil organic matter loss that is symptomatic of long-term warming in temperate forest ecosystems (Bardgett et al., 1999; Schimel and Gulledge, 2004; Frey et al., 2008, 2013). We set out to test the primary hypothesis that the soil bacterial community is altered by warming, and statistically significant differences were only apparent after 20 years. There are many factors in the environment that correlate with warming effects on microbial feedbacks to the climate system, including associated soil moisture and drought, changes in plant communities, and N deposition. For example, Blankinship and colleagues' meta-analysis of 75 manipulative climate change experiments found that warming was more likely to have a negative effect on microbial abundance (density) in cool, dry locations (Blankinship et al., 2011). Observed changes in beta diversity may be due to the loss of labile C (Frey et al., 2008) that represents a significant change in substrate available to resident microbes. Changes in substrate availability in soil are well known to affect changes in microbial community structure (Cleveland et al., 2007; Fierer et al., 2007; España et al., 2011). The extent to which changes in microbial substrate utilization will result in net changes in carbon cycle feedbacks to the atmosphere remain to be examined, and will necessitate an understanding of why communities are changing, as well as how populations' carbon use efficiencies are changing and the extent to which new soil carbon pools (essentially, new niche space) are being degraded by the changing microbial populations.

Our secondary hypothesis, that average bacterial copy number was decreased by long-term warming, was also supported by the ribosomal RNA copy number estimation evidence, showing that that long-term warming favors bacteria with an oligotrophic lifestyle (Klappenbach et al., 2000; Stevenson and Schmidt, 2004). Within a bacterial genome, the number of rRNA gene operon copies tends to correlate with maximum growth rate (Stevenson and Schmidt, 2004), the ability to change growth rates quickly (Klappenbach et al., 2000), and other traits including limited mobility and fewer types of more high-affinity transporters (Lauro et al., 2009), though there are exceptions to these generalizations (Blazewicz et al., 2013). Organisms with many copies of the rRNA gene operon are broadly considered to be copiotrophs, adapted for exploitation of varying and high-quality substrates, while those with single or few copies are considered to be oligotrophs, adapted to extract maximum resources out of a limited supply (Klappenbach et al., 2000; Stevenson and Schmidt, 2004). However, oligotrophy can also occur under conditions where privatization of resources is possible, e.g., conditions of high spatial structure or high heterogeneity (Pfeiffer et al., 2001; Stevenson and Schmidt, 2004; Lennon et al., 2012; Bachmann et al., 2013). It is possible that long-term warming has caused a change in soil structure that has increased the spatial heterogeneity, porosity, or other physical structure of the soils including physical or chemical protection of soil carbon that may contribute to the observed decrease in ribosomal operon copy number, though further study would be required to test this hypothesis.

We assume that the dominant subset of the community likely includes taxa that have important functions in the soil due to their success, though this is based on measures of relative abundance. Our measures of relative abundance based on QPCR suggest that of all bacteria, Actinobacteria comprise 8.8% in control and 9.4% in warmed, that Alphaproteobacteria comprise 25% in control and 44% in warmed soils, and that Acidobacteria comprise 3.4% in control and 7.4% in warmed soils (Table S4). However, the fraction of active populations at any one time may be as low as 10% for soil (Lennon and Jones, 2011), which confounds hypothesized links between function and observed community profiles by soil DNA evidence. Our original hypotheses were that long-term warming would induce a shift in the soil microbial communities, and that observed decreased soil C represented a decrease in microbial substrate availability that would increase the incidence of oligotrophy. These hypotheses are supported by our data, which include increased evenness with long-term warming, decreased ribosomal RNA copy number, increased community evenness and increased relative and absolute abundance of known or suspected oligotrophic taxa.

Ultimately, we are interested in changes in community structure insofar as they can reveal indicators of microbial feedbacks to climate, and because of this, we turned to indicator species and QPCR of key dynamic groups: Alphaproteobacteria, Acidobacteria, and Actinobacteria. Of the Alphaproteobacteria that changed with warming, the Rhizobiales mostly increased with warming while the Rhodospirillales mostly decreased with warming. The Rhizobiales include the most dominant Alphaproteobacteria, an unknown *Hyphomicrobium* spp. (Figure 1C), as well as members that are purple sulfur and non-sulfur bacteria, a group known for being able to grow under a wide range of conditions (Larimer et al., 2004). These taxa contain well-known plant root-associated microbes, though these experimental plots are too small to take into account changes in plant physiology or community, which also affect the observed microbial community in a warmer world (Bardgett et al., 1999). Acidobacteria can grow on complex polymers, including plant hemicellulose or cellulose and fungal chitin (Eichorst et al., 2011). The phyla Alphaproteobacteria, Acidobacteria and Actinobacteria contain many representative taxa known to degrade recalcitrant C and/or that have plant-specific associations (Barret et al., 2011). Further studies may elucidate the genetic or functional differences between the groups thus far represented only by the V4 region sequence and observed changes in relative abundance in a warmer world, though observation of genetic evolution in response to long-term warming or evolved functional changes, such as extracellular enzyme temperature optima, increased tolerance to low water potential conditions, or increased capacity for uptake and degradation of lower quality and quantity carbon will all require measures of physiology of isolated organisms in the lab.

Though absolute copies of Actinobacteria did not differ between warmed and control plots (Figure 3B), increased absolute abundance of Actinobacteria was observed at the Prospect Hill sites by FAME analysis after 12 years of warming (Frey et al., 2008). The most dominant taxon in the control plots, an Actinomycetales (class Actinobacteria, NewOTU5624), decreased 78% in response to warming, declining from 4.5 to 1.02% relative abundance (*P* < 0.05); the second most abundant taxon was also an Actinomycetales (NewOTU189491) and was unaffected by by warming (Figure 1C). In a separate study at Harvard Forest, Actinomycetes were shown to increase in relative abundance with the addition of labile, but not recalcitrant C source (Goldfarb et al., 2011), though these isolates were only incubated with lignin as recalcitrant C for 48 h. There are other studies that suggest Actinobacteria may be a rich reservoir of extracellular peroxidases including lignin peroxidases (Godden et al., 1992; Kirby, 2006), though further studies will determine how far lignin activity among the phylum Actinobacteria extends beyond the streptomycetes (Le Roes-Hill et al., 2011). We hypothesize that the first and second most dominant taxa may be representative of copiotrophic and oligotrophic groups, respectively: their respective estimated copy numbers support this (4.07 for NewOTU5624, and 2.75 for NewOTU189491). This would also explain the net zero change in Actinomycetes by Q-PCR. The reduced amount of labile C in the warmed soils (Bradford et al., 2008) would drive opposing responses of two phylogenetically similar but functionally divergent groups to warming–decreased relative abundance of the potential copiotroph and increase of the potential oligotroph.

Observed changes in fungal biomass were observed by FAME analysis after 12 years of warming at the 20-year warmed site, which showed that warming caused decreased fungal abundance in both the mineral and organic soil horizons (Frey et al., 2008). Reduced fungal biomass has also been observed with warming in other sites (Waldrop et al., 2004; Rinnan et al., 2007; Frey et al., 2008) and though this current study focused mainly on bacteria, the importance of fungi in this systems is being separately studied. Fungi generally dominate primary decomposition in the organic horizon of temperate soils (Berg et al., 1998; Thevenot et al., 2010), and fungal laccase, phenol oxidase and peroxidase activities and genes encoding these enzymes have been found in greater abundance in upper layer, high organic-matter content soils relative to deeper, mineral soils (Luis et al., 2005; Sinsabaugh, 2010). The significant loss in organic horizon soil carbon with long-term warming is likely a combination of increased activity of primary decomposers (generally fungi) in the organic horizon, and increased demand (either through activity or abundance) of secondary decomposers (generally bacteria) in the mineral horizon. Like fungi, Actinomycetes are usually filamentous, and their dominance could suggest changing niches in soil and different contributions to soil C cycling due to long-term warming (Six et al., 2006; De Boer et al., 2008, 2005). Functional analyses of organisms from the organic and mineral horizons separately will suggest mechanisms for the observed substantially depleted soil organic layer mass with warming.

While a community shift was only observed after 20 years, functional changes and thermal acclimation were observed much earlier (Melillo et al., 2002; Bradford et al., 2008; Frey et al., 2013). Shorter term studies have also noticed no change in microbial community by rRNA gene profiles with warming despite increases in respiration, such as temperate mountain forest soil warmed 4°C for 4 years (Kuffner et al., 2012) and mature spruce forest soils warmed 4°C during snow-free seasons for 4 years (Schindlbacher et al., 2011), which is consistent with our observations of the 5-year warmed SWaN plots. Genomic studies such as this permit us to determine whether a change in the community accompanies such changes in function, where no change in community would suggest a high degree of functional redundancy and a capacity for soil community resilience despite the warming treatment (Shade et al., 2012). For example, the KFFL field grassland soils are also dominated by Actinobacteria, Alphaproteobacteria, and Acidobacteria, as are the old-field soils studied by Castro and colleagues, and in both cases, warming alone had a smaller effect than when it was studied in conjunction with elevated CO_2_ (Castro et al., 2010) or with drought (Sheik et al., 2011). This study is valuable in that it permits examination of the long-term effects of warming without confounding environmental and climate factors. The change in community structure that we observed after 20 years suggests that some other aspect of the soil niche space caused pressure on the community, resulting in the change (Schimel et al., 2007). While in some instances a strong positive correlation has been observed between taxonomic and functional richness of bacteria (Konstantinidis and Tiedje, 2007; Richter and Rosselló-Móra, 2009), we expect that expanded functional diversity that has been observed in these soils (Frey et al., 2013) more likely has its origins in changing active populations or genetic adaptation.

The long-term effects of warming, including altered community structure, decreased fungal biomass, increased evenness and decreased ribosomal copy number as an indicator of oligotrophy, all suggest positive warming-induced climate feedbacks. Indications of increased oligotrophy are perhaps the most alarming, because these may suggest that long-term warming is causing decreased physical protection of older or more recalcitrant soil C pools, assuming that physically protected C pools are more recalcitrant and that accessing these pools requires strategies (different enzymes, exploratory growth) consistent with oligotrophy (Schmidt et al., 2011; Bödeker et al., 2014). Testing hypotheses of mechanisms of how warming changes microbial communities, by distinguishing increased enzyme activity from altered enzyme production (Conant et al., 2011), as well as through decreased soil moisture (Toberman et al., 2008; Peñuelas et al., 2012), would benefit from accompanying direct observations on microbial activity, including comparative microbial physiology and genomics of isolated dominant strains. Genomic studies will remain valuable for understanding genomic context for changes in function, and should ultimately enable incorporation of microbial parameters into modeling efforts for prediction of microbial feedbacks to changing climate in a warmer world.

## Conclusions

Our data support the main hypothesis that long-term warming induces changes in microbial community composition, changes that are not seen in intermediate lengths of time. The strong community unevenness and similarity between dominant and whole community beta diversity suggests that a few keystone species may be responsible for a large proportion of the soil C cycling activity. The decreased abundance of dominant bacterial taxa as well as of total fungi (Frey et al., 2008) supports our second hypothesis that there is shifting niche space that may be evidence of changing C availability. The reduced ability of fungi and some Actinobacteria to survive may be due to dwindling resources, shifts in C quality, or a reduction in fine root biomass due to long-term warming, and may have created an opportunity for other oligotrophic bacteria (Butler et al., 2012; Koranda et al., 2013). Understanding the specific contributions of the dominant taxa to soil C cycling would be a powerful tool for modeling the relationship between microbial diversity and changes in climate.

### Conflict of interest statement

The authors declare that the research was conducted in the absence of any commercial or financial relationships that could be construed as a potential conflict of interest.
