# Supplementary material for: Long-term forest soil warming alters microbial communities in temperate forest soils
Source: Front Microbiol. 2015 Feb 13;6:104. doi: 10.3389/fmicb.2015.00104 (PMC4327730; doi:10.3389/fmicb.2015.00104)

DeAngelis et al., 2014

Supplemental Materials

Contents

I. Tables

Table S1. Summary of OTU and observation counts after each successive application of data curation
Table S2. Community differences by groups
Table S3a. Measures of diversity by treatment for each site separately
Table S3b. Measures of diversity by site
Table S4. Quantitative PCR of bacteria and fungi, reported mean copies of small subunit ribosomal RNA gene (copies) per gram soil (dry weight) plus or minus standard error.
Table S5. OTUs of changing abundance in warmed compared to control soils from Prospect Hill site.

II. Figures

Figure S1. Rarefaction curves for each sample.
Figure S2. Ordination for (A) whole community, (B) dominant subset of taxa, and (C) copy number corrected dominant subset of taxa.
Figure S3. Estimated 16S rRNA copy number by relative abundance for the 20-year warmed (Prospect Hill) organic horizon soil microbial community (n=13,847).

Table S1. Summary of OTU and observation counts after each successive application of data curation.

Run	OTUs (count)	Observations (count)	Notes
Total reads	n.a.	11,355,423	Total reads after sequencing
Initial QC	n.a.	10,192,548	Quality filtering and de-multiplexing
Post-assembly	n.a.	5,096,274	Paired-end assembly using FLASH aligner
De-multiplexed	n.a.	3,487,689	Qiime demultiplexed and quality filtered
Open, 99%, raw	108,432	3,145,351	Open referenced OTU calling, no singletons
Open, 99%	86,646	3,020,293	No singletons, chimeras excluded
Open, 99%, no2s	45,875	2,938,751	No singletons or doubletons, chimeras excluded
Open, 99%, no2s, rarefied	43,909	1,561,200	No singletons or doubletons, chimeras excluded, samples rarefied to observations in smallest sample
Open, 99%, >0.1%	155	1,537,364	Dominants (>0.1% relative abundance(>2,938))

Table S2. Community differences by groups for all samples (n=48)

	Organic soil				Mineral soil			
	site	P	treatment	P	site	P	treatment	P
**Full Community (N=45,875)**								
MRPP	0.089	***	0.037	*	0.161	***	0.0194	0.07
Adonis	2.89	***	1.97	*	4.26	***	1.55	0.08
Anosim	0.316	***	0.119	*	0.567	***	0.080	0.09
**Dominant Subset Community (N=155)**								
MRPP	0.091	***	0.041	**	0.175	***	0.023	0.06
Adonis	3.43	***	2.17	*	6.97	***	1.65	0.11
Anosim	0.260	***	0.985	*	0.577	***	0.753	0.10

NB: These results are from tests that were performed on the 99% identity OTUs from the open-reference OTU picking in QIIME, with singletons and doubletons removed and rarefied community. Statistical significance is indicated by * (P<0.05), ** (P<0.01), *** (P<0.001), and *n.s.* (not significant).

Table S3a. Measures of diversity by warming treatment for each site separately for the total community (N=43,909)

	SP (5 yrs)			BW (8 yrs)			PH (20 yrs)		
	Control	Heated	*P*	Control	Heated	*P*	Control	Heated	*P*
Shannon's H	6.22	6.14	n.s.	6.68	6.56	n.s.	6.34	6.56	**
Simpson's D	0.990	0.990	n.s.	0.995	0.994	n.s.	0.991	0.993	0.07
Inverse Simpsons	113.0	112.0	n.s.	206.9	171.8	n.s.	126.6	148.4	0.08
Alpha diversity	1373.4	1267.3	n.s.	1667.8	1555.1	n.s.	1454.2	1599.4	0.06
Richness (S)	4384.0	4149.1	n.s.	5028.1	4795.5	n.s.	4574.5	4893.0	0.06
Evenness (Pielou's J)	0.742	0.738	n.s.	0.784	0.774	n.s.	0.752	0.772	**

NB: Statistical significance is indicated by * (P<0.05), ** (P<0.01), *** (P<0.001), and *n.s.* (not significant).

Table S3b. Measures of diversity by site for the total community (N=43,909)

	**SWaN Plots (5 years)**	**Barre Woods (8 years)**	**Prospect Hill (20 years)**	R^2_{adj}	**F**	***P***
Shannon's H	6.24 a	6.68 b	6.51 b	0.335	12.8	***
Simpson's D	0.9898 a	0.9942 b	0.9924 b	0.265	9.46	***
Inverse Simpsons	112.37 a	189.86 b	136.68 a	0.392	16.1	***
Alpha diversity	1706 a	2015 b	2012 b	0.172	5.89	**
Richness (S)	6101	6835	6992	n.s.		
Evenness (Pielou's J)	0.717 a	0.757 b	0.737 ab	0.27	9.7	***

Table S4. Quantitative PCR of bacteria and fungi, reported mean copies of small subunit ribosomal RNA gene (copies) per gram soil (dry weight) plus or minus standard error. Significance is denoted as * *P*<0.05, *n.s.* not significant. Bayesian inference (*BI*) is also used to compare group sizes, with mean effect size reported and boldface denoting credible differences in control versus heated group sizes. Values for bacteria and fungi are means across all three sites (N=48), while values for the Acidobacteria, Actinobacteria and Alphaproteobacteria are for the Prospect Hill organic soil horizon sites only (N=8).

	Organic					
	Control		Heated		*P*	*BI*
Bacteria	3.88e9	± 9.15e8	4.18e9	± 6.71e8	*n.s.*	0.166
Fungi	1.39e9	± 7.03e8	5.64e8	± 2.12e8	0.07	**0.744**
Acidobacteria	1.32e8	± 1.25e8	3.10e8	± 1.13e8	0.08	**1.31**
Actinobacteria	3.43e8	± 8.81e7	3.94e8	± 8.09e7	*n.s.*	0.691
Alpha-proteobacteria	9.89e8	± 4.45e8	1.84e9	± 1.99e8	*	**2.79**
	Mineral					
	Control		Heated		*P*	
Bacteria	3.57e9	± 7.35e8	3.48e9	± 4.28e8	*n.s.*	0.063
Fungi	4.62e8	± 8.84e7	3.79e8	± 7.83e7	*n.s.*	0.475

Table S5. OTUs of changing abundance in warmed ("W") compared to control ("C") soils from Prospect Hill site. Tests performed were T1, IndVal; T2, volcano; T3, NSC; T4, pls-da; T5, Bayesian groups; T6, Rank abundance T7, Student T-test uncorrected; see Methods for further details about the tests performed.

Phylum	Subphylum Taxonomy	OTU	Rel Abund Control (%)	Rel Abund Warmed (%)	Ratio C:W	T1	T2	T3	T4	T5	T6	T7	Sum of Significance Tests
Acidobacteria	Acidobacteria; Acidobacteriales	NewOTU232430	0.073	0.388	0.188	W	W				W		WWW
	Acidobacteria; Acidobacteriales	NewOTU321566	0.523	1.138	0.460	W	W						WW
	Acidobacteria; Acidobacteriales	NewOTU402783	0.061	0.125	0.488		W						W
	Acidobacteria-2	NewOTU216724	0.075	0.363	0.208	W	W				W		WWW
	Acidobacteria-2	NewOTU90931	0.053	0.247	0.215	W	W					W	WWW
	Acidobacteria-2	NewOTU17822	0.069	0.281	0.246	W	W						WW
	Acidobacteria-2	NewOTU373042	0.403	1.132	0.356	W	W						WW
	Acidobacteria-2	NewOTU216750	0.018	0.179	0.103						W		W
	Acidobacteria-2	NewOTU85375	0.017	0.074	0.229		W						W
	Acidobacteria-2	NewOTU331926	0.014	0.059	0.234	W							W
	Acidobacteria-2	NewOTU337407	0.042	0.127	0.333		W						W
Actinobacteria	Actinobacteria; Actinomycetales	NewOTU241326	0.026	0.127	0.206	W	W						WW
	Actinobacteria; Actinomycetales	1075732	0.245	0.871	0.282	W	W						WW
	Actinobacteria; Actinomycetales	NewOTU72804	0.062	0.155	0.401		W						W
	Thermoleophilia; Solirubrobacterales	NewOTU379583	0.119	0.249	0.478		W						W
Planctomycetes	Planctomycetia; Gemmatales	NewOTU285592	0.021	0.174	0.119	W							W
	Planctomycetia; Planctomycetales	938844	0.025	0.123	0.200		W						W
Proteobacteria	Alpha-; Rhizobiales	NewOTU362740	0.019	0.105	0.184	W	W				W		WWW
	Alpha-; Rhizobiales	NewOTU393214	0.142	0.753	0.189	W	W			W			WWW
	Alpha-; Rhizobiales	NewOTU196341	0.025	0.142	0.173	W	W						WW
	Alpha-; Rhizobiales	545247	0.126	0.445	0.283	W	W						WW
	Alpha-; Rhizobiales	NewOTU431587	1.782	2.760	0.646	W		W					WW
	Alpha-; Rhizobiales	NewOTU393499	0.080	0.259	0.309	W							W
	Alpha-; Rhodospirillales	NewOTU211289	0.029	0.193	0.151	W							W
	Delta-; Syntrophobacterales	NewOTU390196	0.045	0.178	0.251	W	W						WW
	Delta-; Syntrophobacterales	NewOTU307821	0.021	0.059	0.351		W						W
	Gamma-; Xanthomonadales	NewOTU196148	0.045	0.228	0.195	W	W						WW
Acidobacteria	Acidobacteria; Acidobacteriales	NewOTU197652	0.668	0.435	1.535	C							C
Actinobacteria	Actinobacteria; Actinomycetales	NewOTU5624	4.493	1.018	4.412	C	C	C	C			C	CCCCC
	Actinobacteria; Actinomycetales	NewOTU151494	0.667	0.062	10.716	C					C		CC
	Actinobacteria; Actinomycetales	NewOTU88918	0.154	0.044	3.509						C		C
Proteobacteria	Alpha-; Rhizobiales	NewOTU162828	0.337	0.788	0.428		C						C
	Alpha-; Rhizobiales	NewOTU126606	0.028	0.058	0.493						C		C
	Alpha-; Rhizobiales	NewOTU323079	0.367	0.152	2.409	C							C
	Alpha-; Rhodospirillales	NewOTU164307	0.380	0.051	7.485	C				C	C		CCC
	Alpha-; Rhodospirillales	NewOTU357934	0.430	0.201	2.137	C	C						CC
	Alpha-; Rhodospirillales	NewOTU33857	0.313	0.099	3.155	C							C
	Gamma-; Xanthomonadales	NewOTU327286	1.041	0.707	1.472	C							C
	Gamma-; Xanthomonadales	NewOTU230354	0.425	0.209	2.033	C							C

Figure S1. Rarefaction curves for each of the 48 samples from the Harvard Forest long-term warming experiment.

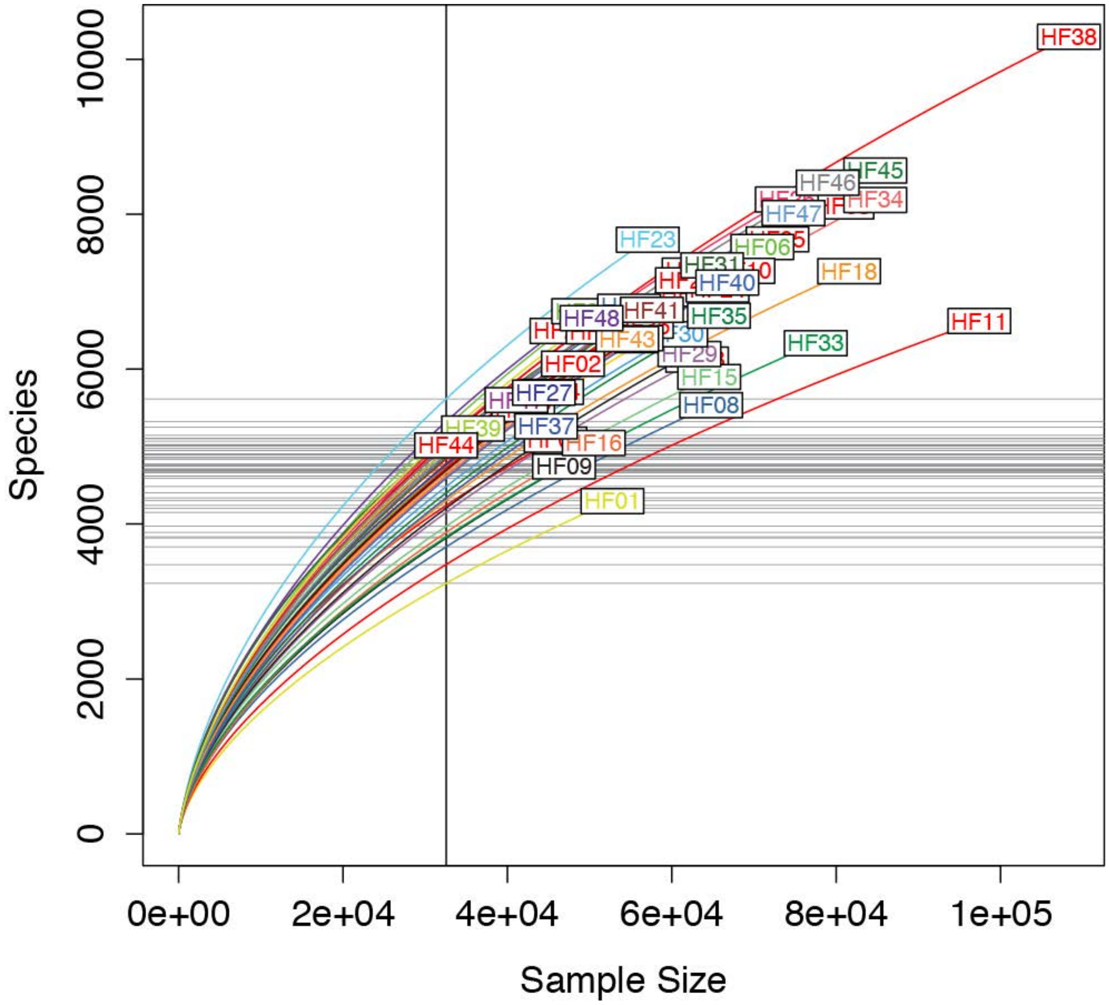

Figure S2. Ordination for (A) whole community, (B) dominant subset of taxa, and (C) copy number corrected dominant subset of taxa.

A. All samples, no singletons or doubletons (N=43,909)

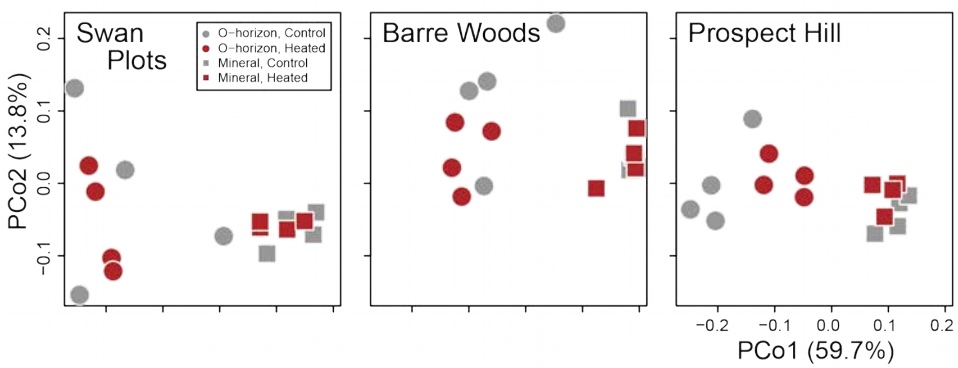

B. Dominant OTUs only, >0.1% relative abundace (N=155)

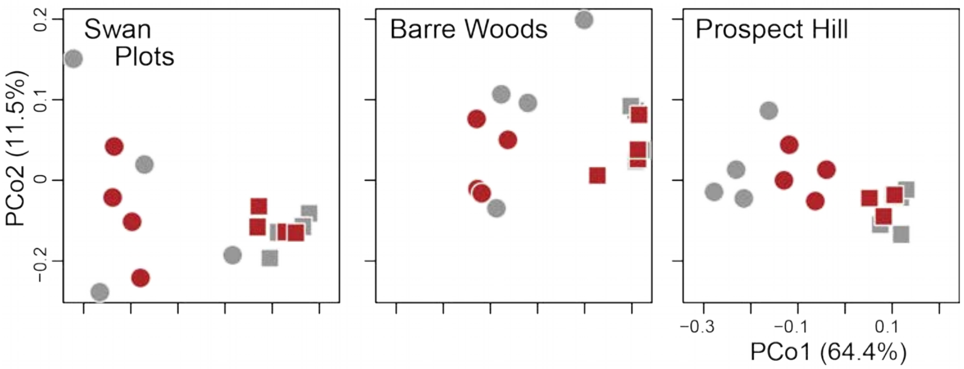

C. Dominant OTUs only with copy number corrected (N=155)

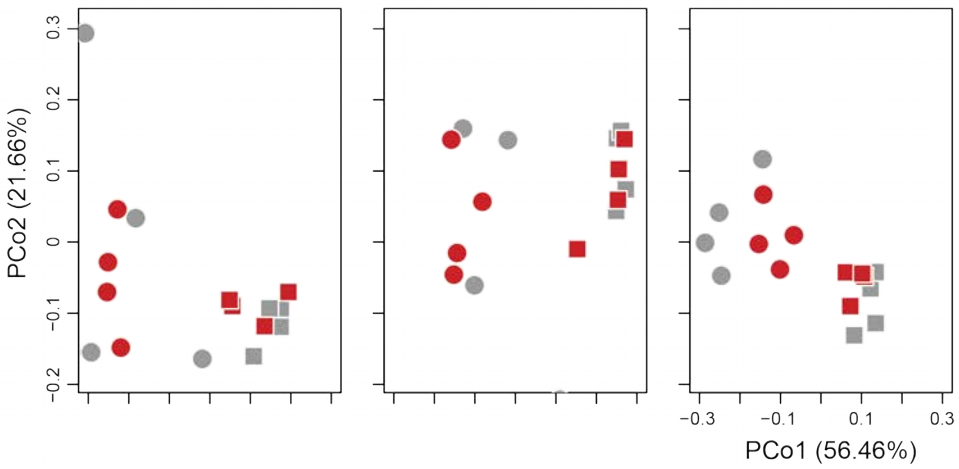

Figure S3. Plot of estimated 16S ribosomal RNA copy number by relative abundance for the dominant subset community (n=155) in the Prospect Hill (20-year warmed) organic horizon soil microbial communities. Estimated 16S ribosomal RNA copy number is calculated based on phylogenetic inference; see methods for details. While the most abundant taxa (the top 15) in these forest soils tend to have only a few copies of the 16S rRNA operon, taxa that ranked in the top 155 (but not the top 15) tended to have more copies than average and would have been overestimated in their abundance

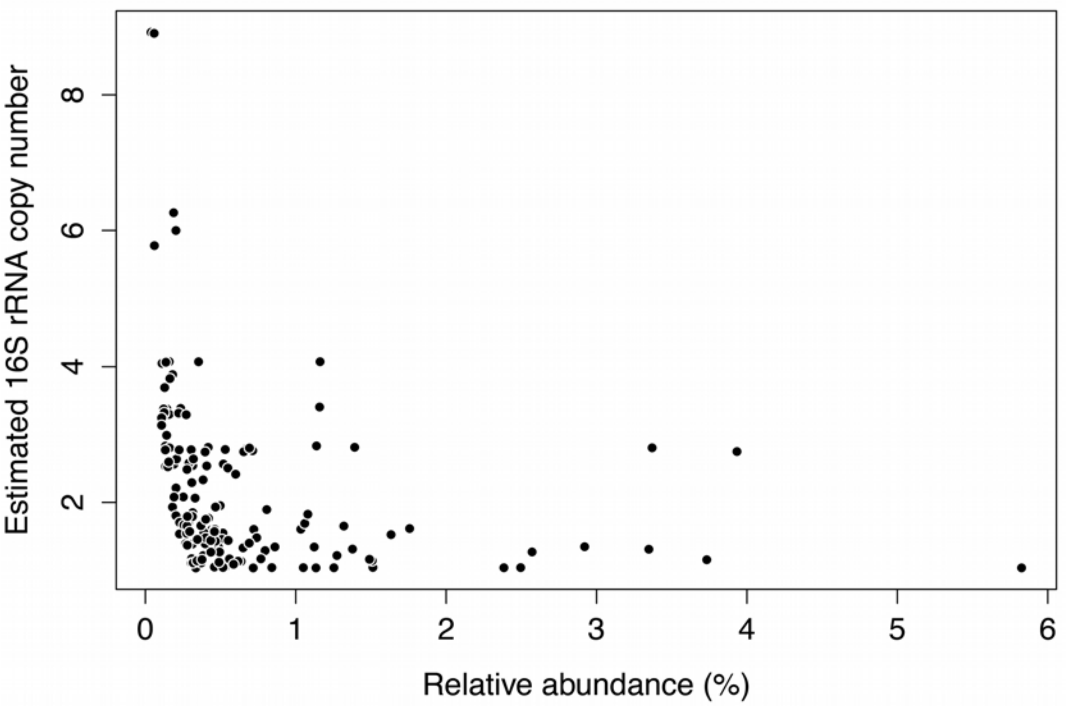

Supplement: Supplementary file 1 [file Presentation1.PDF]
